# Absolute Radiometric Calibration of ALS Intensity Data: Effects on Accuracy and Target Classification

**DOI:** 10.3390/s111110586

**Published:** 2011-11-07

**Authors:** Sanna Kaasalainen, Ulla Pyysalo, Anssi Krooks, Ants Vain, Antero Kukko, Juha Hyyppä, Mikko Kaasalainen

**Affiliations:** 1 Department of Remote Sensing and Photogrammetry, Finnish Geodetic Institute, Geodeetinrinne 2, P.O. Box 15, 02431 Masala, Finland; E-Mails: anssi.krooks@fgi.fi (A.K.); ants.vain@emu.ee (A.V.); antero.kukko@fgi.fi (A.K.); juha.hyyppa@fgi.fi (J.H.); 2 Department of Geoinformatics and Cartography, Finnish Geodetic Institute, Geodeetinrinne 2, P.O. Box 15, 02431 Masala, Finland; E-Mail: ulla.pyysalo@fgi.fi; 3 Estonian University of Life Sciences, Tartu 51014, Estonia; 4 Department of Mathematics, Tampere University of Technology, P.O. Box 553, 33101 Tampere, Finland; E-Mail: mikko.kaasalainen@tut.fi

**Keywords:** LiDAR, 42.68.Wt, 42.79.Qx calibration, 06.20.fb remote sensing, 07.07.Df 07.07.Df sensors, remote sensing

## Abstract

Radiometric calibration of airborne laser scanning (ALS) intensity data aims at retrieving a value related to the target scattering properties, which is independent on the instrument or flight parameters. The aim of a calibration procedure is also to be able to compare results from different flights and instruments, but practical applications are sparsely available, and the performance of calibration methods for this purpose needs to be further assessed. We have studied the radiometric calibration with data from three separate flights and two different instruments using external calibration targets. We find that the intensity data from different flights and instruments can be compared to each other only after a radiometric calibration process using separate calibration targets carefully selected for each flight. The calibration is also necessary for target classification purposes, such as separating vegetation from sand using intensity data from different flights. The classification results are meaningful only for calibrated intensity data.

## 1. Introduction

### 1.1. Background: ALS Intensity and Its Application in Remote Sensing

Airborne laser scanning (ALS) is an active remote sensing system that mostly uses monochromatic, pulsed lasers to measure the surface topography and object characterization [1]. ALS is based on Light Detection and Ranging (LiDAR) range measurements between the laser sensor and the target, and results in a point cloud (*x*, *y*, *z*) representing the coordinates of the object. The intensity (*I*) value is most often recorded for each point as well. The intensity represents the measured power or amplitude of the received pulse or waveform. The basic idea in the radiometric calibration is to correct the returned pulse power or waveform amplitude into parameters equal or proportional to the target scattering properties.

The use of (uncalibrated or calibrated) ALS intensity data has enhanced the development of automatic methods for point selection and object classification from point clouds. There are applications such as detection of tree species and estimating the leaf area index (LAI) or canopy structure (e.g., [2]), and studying the pulse intensity return ratio to analyze canopy structures and fractional cover [3]. While most of these studies operate in near-infrared (NIR) wavelengths, it has also been demonstrated that the green laser return intensity is correlated with the chlorophyll content in broadleaf canopies [4]. Combining the intensity with point cloud data has provided quick and easy method for classification of land cover types and estimating biomass [5]. One example is the analysis of mixed-species plantation, where the LIDAR intensity provided a useful predictor of the proportion of different mixtures of conifer species [6]. Intensity is also used as discriminator of ground/non-ground points in generating digital terrain models (DTM) [7].

ALS intensity data have also been increasingly applied in glaciology, e.g., for separating different target classes [8], and in ocean or coastal studies, where the intensity data improves the classification accuracy, e.g., of water points or habitat and stands for vegetation species [9,10]. It is also possible to use intensity data for separating vegetation from other targets in land cover classification [11], but extra information from other data sources is needed for more detailed classification. Dual wavelength lasers have been used in bathymetry and studies of coastal ecology, where the intensities at both wavelengths can be used to calculate spectral indices, such as the normalized difference vegetation index (NDVI) [12]. Intensity data are also useful in combining ALS point clouds with digital images or matching data from different standpoints [9,13]. A new field of interest in the remote sensing community is also the fusion of LIDAR and radar data (e.g., [14,15]). Intensity variations have a strong role in these studies and there is potential for further applications.

### 1.2. Radiometric Calibration of ALS Intensity

As the interest in the radiometric calibration has increased during the recent years, a great number of new studies on the methodology have been published in the past 1–2 years only. The first radiometric calibration methods and their applications have been presented in [16–20]. Physical concepts of laser scanner intensity calibration are reviewed in, (e.g., [19,21]).

The recently published radiometric calibration efforts have introduced different approaches to the intensity correction. External reference targets have been used as reflectance standards [17,20,22–24], for which the reflectance has been measured either *in situ* or in the laboratory. There have also been validation efforts based on simultaneously acquired data from other (passive) remote sensing instruments [25]. Calibration for full waveform ALS has been investigated in [18,21], and practical applications have recently become available [23,26]. One of the newest topics is the effect of automatic gain control (AGC) used in some instruments, which is intended to optimize the range measurement, but calls for normalization in order to make the intensity data useful [27,28]. Procedures for radiometric calibration have also been recently presented for terrestrial laser scanning (TLS), but only few studies are thus far available [29–31].

Since intensity is affected by the flight and laser parameters together with the object properties, the effect of flight parameters has to be calibrated. This is often called relative calibration. The idea is to normalize the measurements from different altitudes, incidence angles, and dates, so that they are comparable for the same system, *i.e*., in a single sensor case. The relative calibration methods are mostly based on modelling the intensity or backscatter cross section in terms of the radar equation [19], or normalizing the instrumental intensity into a standard range to remove the path length variations and range dependence of the intensity signal [5,6,32]. Normalization with respect to range and incidence angle enables the fusion of data from different flight paths [33].

Absolute radiometric calibration aims at producing intensity values that are independent on, e.g., the atmospheric and other non-instrumental effects, and depend only on the target scattering properties. This is necessary for combining multi-temporal data from different sensors and flight altitudes and for the comparison of data from different sources. Most of the radiometric calibration studies thus far focus on a single sensor only (and mostly a single flight—although there are studies that combine data from multiple flight lines or days [5,23,34]). Furthermore, more studies are needed to evaluate the general outcome of the radiometric calibration, *i.e.*, whether it is actually capable of producing results that are comparable between different experiments. The calibration has been found to be a challenging task, and still requires further study, especially for accuracy evaluation for multi-temporal and multi-sensor cases, and to correct for other errors [23,35].

### 1.3. The Scope of This Article

Thus far, calibration efforts have mostly only focused on a single sensor, and therefore it is not quite well established whether the intensity data from different sensors or campaigns can actually be combined or used for classification. Our study involves data from three separate flights with two different instruments. The aim of this study is to evaluate the reliability of radiometric calibration and its capability of producing accurate (backscatter) reflectance values. With a bulk of field targets investigated during the study, we also study the effect of calibration on target classification, even though the intensity scale has been significantly limited in this study. This is, however, the case in many discrete return ALS campaigns, as the intensity is optimized for the range measurement. Knowing the capabilities and limits of the use of intensity data is important in these applications. There is also a great need for further investigations for well-established procedures.

The paper is organized as follows: Section 2 describes the physical background of ALS intensity calibration. The data correction procedures, data acquisition (*i.e.*, the flight campaigns), and the application of the radiometric calibration procedure to these data have been outlined in Section 3. The results and discussion are presented in Section 4. Section 5 provides the conclusions.

## 2. Physical Background for ALS Intensity

This section presents concisely the physical basis of radiometric calibration of ALS intensity. More detailed reviews of the physical concepts are found in [18,19,21].

The radar equation defines the received power as a function of sensor parameters, and it has also been applied in laser scanning. The equation defines the power entering the LIDAR receiver as [18]:

(1) $$P_{r}=\frac{P_{t}D_{r}^{2}}{4\pi R^{4}\beta_{t}^{2}}\sigma$$

where *P_t_* is the transmitted power, *D_r_* is the receiver aperture, *R* is the range, and *β_t_* is the transmitter beam width. The backscatter cross-section *σ* describes the scattering of a wave from an object, and depends on the scattering solid angle *Ω*, the target area illuminated by the laser beam *A_i_*, and the reflectivity *ρ* [21,23]:

(2) $$\sigma =\frac{4\pi }{\Omega }\rho A_{i}$$

The cross-section per unit illuminated area is expressed as:

(3) $$\sigma^{0}=\frac{\sigma }{A_{i}}$$

As the illuminated target area *A_i_* changes with the angle of incidence, the surface scattering is related to the cross section of the incoming beam *A_i_* cos*Θ_i_* (*Θ_i_* being the angle between incident laser beam and the surface normal):

(4) $$\gamma =\frac{\sigma }{A_{i}\text{cos}\;\theta_{i}}$$

where *γ* is the bistatic scattering coefficient (see [21] for more details). Some of the most recent calibration schemes are based on the retrieval of either *σ* or *γ* as a calibration parameter of full waveform ALS [21,23,24].

In practice, the laser scanner detectors measure the flux of photons entering the receiver from a given direction and solid angle, *i.e.*, the scattered radiance, which is related to the power of the received signal. The discrete return laser scanners record the power of the received pulse as a single digital number representing the intensity. The recorded intensity is proportional to the bidirectional reflectance distribution function (BRDF) of the target, which can be written in terms of laser power as [35]:

(5) $$\text{BRDF}=\frac{\partial^{2}P_{s}}{\partial P_{i}\partial \Omega_{S}}\frac{1}{\text{cos}\Theta_{s}}$$

where *P_s_* and *P_i_* are the scattered and incident power, respectively, *Ω* is the scattering solid angle, and *Θ_s_* is the angle between the scattering direction and the surface normal. In fact, most commercial reflectance spectrometers measure the (directional) hemispherical reflectance (*i.e.*, albedo), which is the total fraction of the incident collimated power on unit surface area scattered into upper hemisphere by unit area of surface [36]. Laser scanners measure only the fraction of the hemispherical reflectance that occurs in the direction of illumination (0° angle between light source and detector), which can be approximated by the BRDF at *Θ_s_* *= Θ_i_*, which we here call the backscattered reflectance. This quantity describes the backscatter properties rather than the reflectance into the entire hemisphere, and even though these two are related, there are other factors that affect the backscatter of an object, which are related to the physics of the surface and the scattering geometry (see [37] and refs. therein). As a practical approach, the backscattered reflectance has been used as a calibration parameter for discrete return LiDAR intensity (*cf.* [20,38]). The results are comparable to laboratory and reference measurements (see the next section), although the applicability for, e.g., targets with multiple echoes has to be further assessed. The method also requires the calculation of the incidence angle (*cf.* [21]).

The “backscattered reflectance” is used in this paper as a calibration parameter. It has been measured as the laser power of the target calibrated with the similarly measured laser power of the reflectance standard (*cf.* [36]). Although it is practically impossible to find a perfect Lambert reference (which are used in, e.g., reflectance spectroscopy) for ALS applications (mostly because of the size requirements for field use of these targets [20]), it is possible to determine the reflectance of the field standards *in situ* or in laboratory using a practically Lambertian standard, and then use these pre-calibrated field standards to calibrate the ALS intensity. The practical procedure is explained in more detail in Section 3.

## 3. Methods

### 3.1. The Calibration Procedure

**Figure 1.**
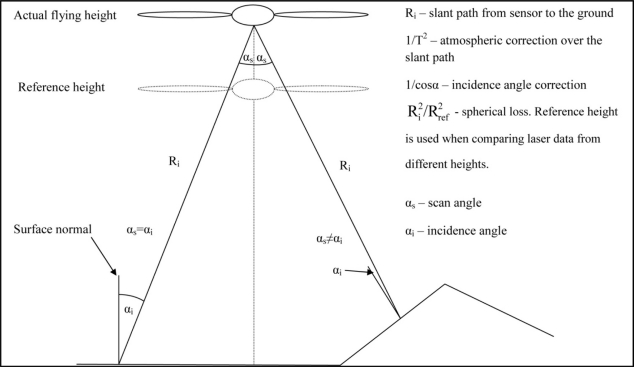
The incidence angle and scan angle. Some of the atmospheric correction parameters are also shown (*cf.* Equation (6) and [34]).

The calibration method used in this study has been developed for discrete return ALS and TLS intensity calibration, based on pre-calibrated *in situ* reference targets placed within the laser scanner field of view. For commercial and naturally available targets, the backscattered reflectance is determined by reference measurements either *in situ* using a near-infrared (NIR) digital camera, or in laboratory [17,20,34,38]. The intensity values (raw data) from the ALS point cloud are extracted using the TerraScan (TerraSolid Ltd.) software, and relatively corrected using the following equation [17,19]:

(6) $$I_{\mathrm{corrected}}=I_{\mathrm{original}}\cdot \frac{R_{i}^{2}}{R_{\mathrm{ref}}^{2}}\cdot \frac{1}{\text{cos}\alpha }\cdot \frac{1}{T^{2}}\cdot \frac{E_{\mathrm{Tref}}}{E_{Tj}}$$

where *I_original_* is the raw intensity value, *R_i_* is the slant distance from the sensor to the ground, *R_ref_* is the chosen reference height, α is the incidence angle (same as the scan angle for horizontal surfaces, see Figure 1), *T* is the total atmospheric transmittance, *E_Tref_* is the reference pulse energy value, and *E_Tj_* is the pulse energy value in the flight line. The reference range is needed for comparing flights at different altitudes; usually one of the flight ranges is chosen. The atmospheric effect (*1/T^2^*) is modeled using the MODTRAN (ver. 3) software (see [34] for more details on the procedure).

The pulse energy *E_Tj_* is related to the pulse repetition frequency (PRF). The initial pulse energy must be high enough that the receiver detects the returning pulse. An increase in flying altitude also increases the path length, resulting in a longer travelling time for the pulse. This usually causes a decrease in the PRF, and the pulse energy must be increased because of the additional attenuation introduced by the increased path length. The pulse energy and its changes are not recorded or reported by most laser scanner manufacturers. Therefore it is usually omitted in the calculations, or cancelled out when external reference targets are used and located in the same flight line. The calibration for different pulse energies is discussed in [17,19]. Pulse energy values for Optech scanners at some PRF settings are reported in [39].

Using reference targets of known or pre-calibrated reflectance, the backscattered reflectance *R_Backscatter_* of the target (the absolute calibration) can be expressed as (*cf.* [36] formula for target hemispherical reflectance):

(7) $$R_{\mathrm{Backscatter}}(\mathrm{target})=\frac{I_{\mathrm{corrected},\mathrm{target}}}{I_{\mathrm{corrected},\mathrm{reference}}}R_{\mathrm{Backscatter}}(\mathrm{reference})$$

where *I_corrected,target_* and *I_corrected,reference_* are extracted from the point cloud data and corrected similarly for both the target and reference, according to Equation (6). *R_Backscatter_(reference)*, *i.e*., the (backscattered) reflectance of the reference target, is obtained from reference measurements. (The reflectance values have no units, see, e.g., [36]).

### 3.2. The Flight Campaign

**Figure 2.**
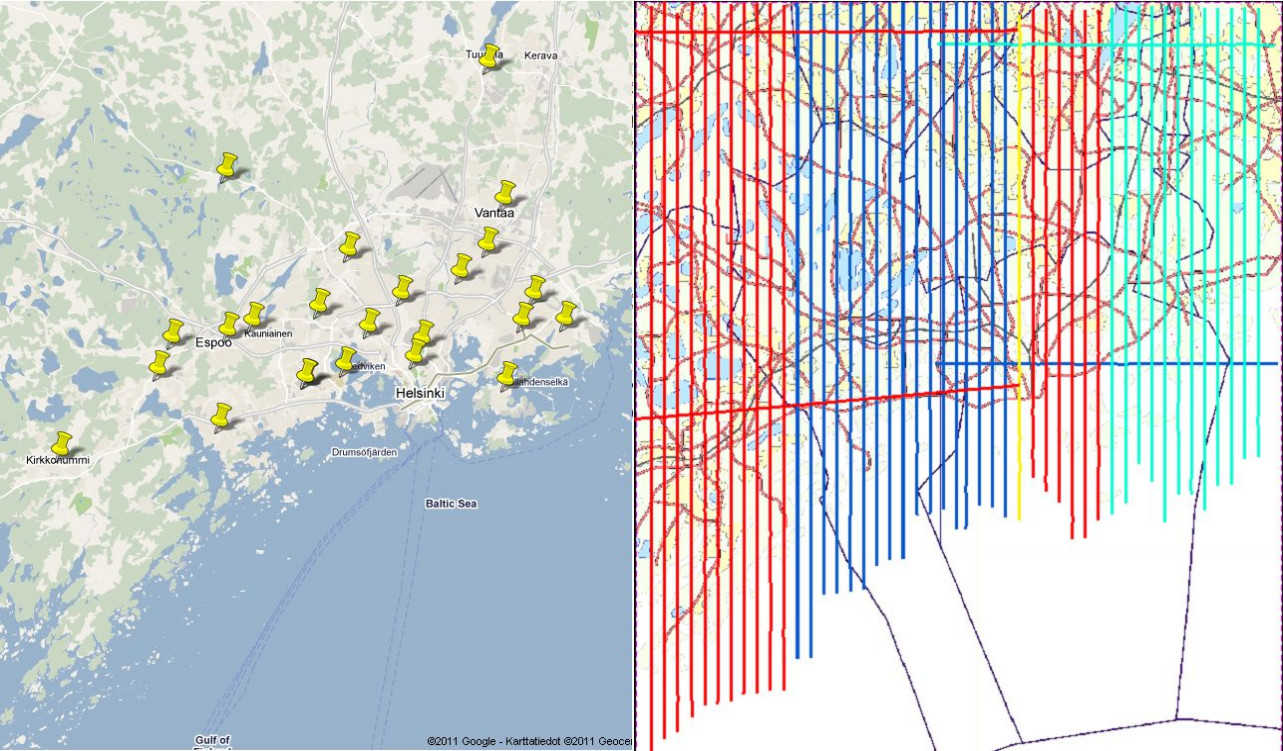
**(a)** Left: the Helsinki metropolitan area and the reference target locations. For easier comparison, similar land cover types were chosen around the entire scanning area, such as football fields, sand fields, beaches and golf courses. The width of the area is about 45 km. (Map © 2010 Google, Map Data © 2010 Geocentre consulting); **(b)** Right: the ALS flight lines shown over the same area. The blue, red (middle), and bluish green lines denote the 9 April, 13 May, and 12 May flights, respectively. The scanned area is about 2,500 km^2^ and a typical length of a flight line is 45 km (shorter in the east because no measurements have been made over the sea). (Map © Maanmittauslaitos 53/MML/10.)

The ALS data analyzed in this study were collected at three separate flights in 9 April and 12–13 May 2008 in the Helsinki capital area (Figure 2).

The campaign was organized by the National Land Survey of Finland as a part of the production of a new national elevation model. The flights were operated by Blom Kartta Oy. Two different Optech ALTM Gemini laser scanners (1,064 nm) were used, one in Apr 9 and the other in 12–13 May. The scanned area consisted of 52 flight lines in 2,500 km^2^ area, and the flying altitude was 1,900 meters with a 0.5 points/m^2^ point density. As the main purpose was to produce an elevation model rather than to investigate the intensity, there were practically no overlapping regions in these flights. The data were recorded in LAS 1.0 format (see http://www.asprs.org/Standards/ for more information) and the intensity capture occurred in 12-bit dynamic measurement range, but only a small part of the range was actually used: the intensity raw values varied between 0–150 counts (sensor units) instead of 0–4,095 counts allowed by the 12-bit format. This means that the measurement range for the intensity was greatly reduced from what was originally possible for the instrument, and it was therefore not possible to bring out any small differences (such as those between different types of vegetation) in the intensity data. The intensity data extraction and correction were carried out according to the procedure described in Section 3.1, including corrections for range and atmospheric transmission. Since the intensity samples were extracted from mostly flat and horizontal surfaces, the angle correction was based on the scan angle. The sample size was approximately 100 m^2^. The intensity values were averaged over all points inside the sample. The typical error of the average intensity value, in terms of standard deviation, was about 13%–15%. This seems to be a typical error range for ALS intensity data (*cf.* [20,34]).

### 3.3. Ground Reference

**Figure 3.**
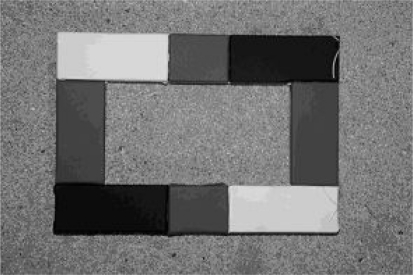
*In situ* reference measurements: a calibration image of the Fuji IS PRO near-infrared digital camera showing the target and the reference frame. The size of the frame is 21 × 29.5 cm (*i.e.*, about the size of an A4 paper).

A near-infrared (NIR) digital camera system consisting of a Fuji IS PRO digital camera and Nikon SB800 electronic flash were used for the *in situ* reflectance measurements of the calibration targets. The output power variation of the flash was ±2%, providing a constant illumination [30]. The backward measurement direction was achieved by mounting the camera on a tripod with the lens facing downwards to the target. A calibration frame with 80%, 19%, and 5% reflectance coatings was placed in the image field so that the target was photographed inside the frame (Figure 3). In this experiment the 19% reflectance (middle gray) panel was used in the calibration. Middle gray targets are commonly used in photography to calibrate light meters, so it was an optimal target for camera calibration, as it was also best related to the reflectance level of many field targets. The sensitivity of the camera was 350 to 1,200 nanometers, but a 1,000 nm IR-filter was used to restrict the wavelength range close to the ALS wavelength.

As the scanned area was large, reference images were collected from a large area as well (see Figure 2 showing the reference target locations). We selected naturally available field targets that were easily accessible (except for some targets such as golf greens and bunkers, for which permission was needed) and had shown stability and good results in our previous studies [20,34]. The images were mostly taken during the summer 2009 and spring 2010, preferably in similar conditions (weather/humidity *etc*.) as the flight. Five camera exposures were taken and averaged from each target. The 14-bit raw format images (ISO100, 1/250 s exposure time) were exported into linear 16-bit tagged image file format (TIFF), which means that the intensity was measurable in a scale from 0 to 2^16^ = 65,536 counts. The images were corrected using a flat field image, and the intensity values were recorded from the green channel. The average error for the camera measurements in terms of the standard deviations of 5 exposures has been measured to be about 2.5% (see [30] for more details).

### 3.4. Applying the Calibration Procedure

The flight lines of different flights are shown in Figure 2. The orange flight lines on the left were also flown on 12 May, but no targets are included because the intensity levels did not match with the other 12 May flight lines. As there was a clear difference between intensity levels at different blocks of flight lines (Figure 2), it was not possible to calibrate the intensity with one reference target from the entire area, but a separate calibration target had to be used for each day. The list of targets for each flight is shown in Table 1, where the calibration targets for each day are marked as boldface. All targets in 9 April were calibrated with Laurinlahti sand field, 12 May with Tapanila football field, and 13 May with Kallahti beach target. The reflectance measured with the Fuji camera was taken as a reference value for the calibration targets. The ALS intensity of the other targets from the same day were calibrated according to Equation (7), where *I_corrected,target_* and *I_corrected,reference_* were taken from ALS uncalibrated data, and *R_Backscatter_(reference)*, was the backscattered reflectance of the calibration target, measured with the Fuji camera system.

## 4. Results and Discussion

**Figure 4.**
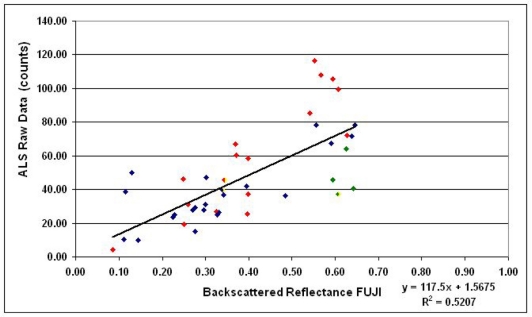
Uncalibrated ALS intensity data from all three flights, plotted against the reference data (backscattered reflectance) measured with the Fuji camera. 9 April, 12 May, and 13 May data are marked as blue, green, and red, respectively. The values of calibration points for each flight are denoted with yellow.

**Figure 5.**
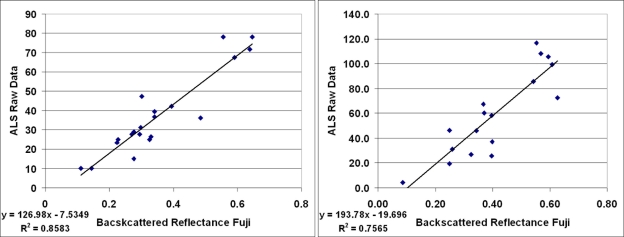
Uncalibrated ALS intensity data from 9 April (left) and 13 May (right) flights, plotted separately against the reference data.

**Figure 6.**
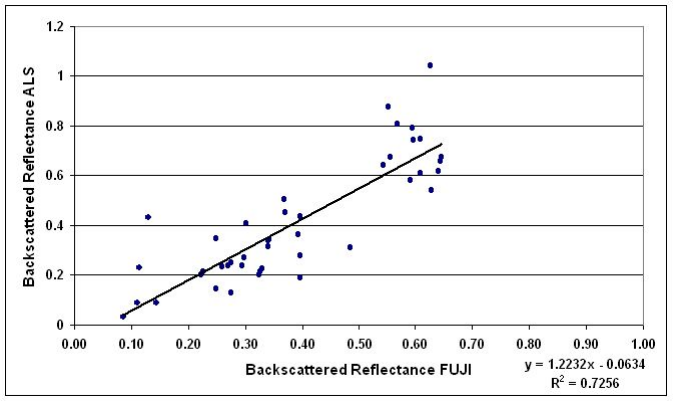
Calibrated ALS intensities for all the targets (all flights) plotted against the Fuji reference data.

Table 1 lists all the samples from each flight and shows their backscattered reflectance values measured from ALS data and Fuji images. Altogether 43 samples were included in the analysis. The intensity values from ALS and Fuji have also been plotted in Figures 4–6 where the results are shown and discussed. For some vegetation targets, the images were taken at entirely different stage of the growing season than ALS, which might have caused uncertainty, so many of the grass/vegetation targets have not been included. In any case, deviation and random errors are always present in ALS intensity data. This has been the case in our previous ALS studies as well [34,38]. The uncalibrated ALS intensity (raw) data are compared with the reference data measured with Fuji in Figure 4, where data from all three flights have been combined.

The correlation coefficient R^2^ of the uncalibrated ALS (intensity values corrected relatively according to Equation (6)) and Fuji digital camera results was 0.52, as seen in Figure 4. The corresponding correlation values of the intensities plotted separately from the two flights were 0.86 and 0.76 for 9 April and 13 May, respectively (see Figure 5). This indicates that the ALS intensity is better consistent with reference values within the same flight. To investigate whether the radiometric calibration would improve the correlation, a similar plot is presented for calibrated ALS data (*i.e.*, the backscattered reflectance) in Figure 6. The correlation coefficient R^2^ between calibrated ALS and Fuji reference data increased from 0.52 to 0.73, which means that intensities from different flights have become better comparable. Particularly, the slope and intercept of the trend line in Figure 6 are close to 1.2 and 0.0, which indicates a reasonable accuracy of the reflectance calibration, while the poor precision, *i.e.*, deviation (scatter) and random errors in the measured intensity may decrease the correlation coefficient.

The radiometric calibration improved the correlation between ALS and reference intensities, but the following practical aspects were also observed from the results:

- There is large variation (*i.e.*, errors and deviation) in the intensity data produced by most (discrete return) ALS instruments. This was also true for the intensities analyzed in this study. Some of these errors may be caused by the limited measurement accuracy or power level variations of the instrument, because the intensity is related to the received power defined at the triggering moment. There are also some external effects, such as a specular reflection from a target (such as a lake surface). Nevertheless, the lack of stability in ALS intensity data (*i.e.*, there is variation in the laser echo amplitudes, see, e.g., [20] and [23]) will always introduce error to the results, such as a large standard deviation of the average intensity.
- There were clear differences in (uncalibrated) intensity levels between each flight dates, which could also be seen in the entire data block. Even the intensity levels between 12 and 13 May, obtained with the same instrument, did not coincide. Therefore, combining intensity data from different flights (or flight dates) is not possible without radiometric calibration.
- Even the intensity levels during the same day did not match: the orange lines in 12 May were entirely different from those in the bluish green flight lines. This means that the calibration target has to be located along the same flight line as the actual targets.
- Choosing a target for calibration is not easy because of the deviation and errors in the intensity data. The raw intensity (counts) should be in the level that best corresponds with the target reflectance, *i.e.*, the ALS measurement must been successful enough to represent the target reflection properties. This is not the case for any target. Intensity measurements from several ground reference (Fuji data) points are necessary to be able to study the stability of data. In this paper, the stability is assessed from the correlation between reference (Fuji) and (uncalibrated) ALS intensity. This will improve the statistics and the selection of a more reliable reference.
- For the 9 April and 13 May flights, targets with intensity values near the regression trend line (Figure 4) were chosen. Only four targets were available for 12 May, so it was more difficult to judge the stability. As the ALS intensity levels appeared lower in 12 May than the other two flights (this was also seen in the entire block of ALS data), one of the targets with lower counts was chosen (see Figure 4). Much worse results in terms of correlation coefficient, slope, and intercept were obtained by choosing an unstable calibration target (*i.e.*, a target with large discrepancy between ALS and Fuji data).
- A greater number of reference targets would still be needed to decrease the level of uncertainty, but that is mostly not possible in practice. Collecting field data is labour intensive and time consuming, so the results have to be obtained with a reasonable number of points.

**Figure 7.**
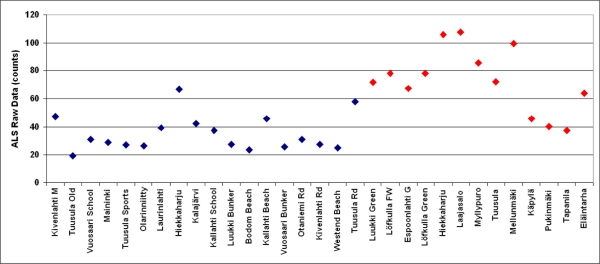

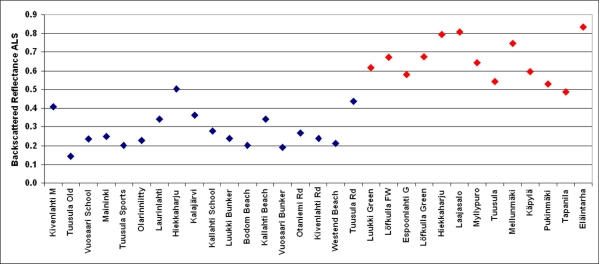
Separating different target types from uncalibrated (top) and calibrated (lower) intensity: ALS intensity/backscattered reflectance for sand (blue points) and grass targets (red points). A difference in the reflectance levels becomes more evident for the calibrated intensity data, and there is a clear improvement in the comparison results using the Student’s t-test.

To test the feasibility of separating different types of targets within the compressed dynamic range (only up to 150 counts in this study), we plotted the intensity data for sand and grass targets (in Table 1) before and after calibration in Figure 7. After calibration, the results are more clearly divided into two groups by their backscattered reflectance value. The grasses appear more reflective than the sands, and the overlap between sand and grass reflectance values has decreased. Using Student’s t-test (which is the most suitable one for this kind of comparison essentially based on the means of datasets), we find that the calibrated datasets of sand (measured in 9 April and 13 May) are drawn from distributions with the same mean with 90% probability, whereas uncalibrated sand data have the same mean with only 40% probability. For the grasses (9 April and 12–13 May), the calibrated data correspond to the same mean with 70% probability, and the uncalibrated data do so only with 20% probability. We thus conclude that not only are the probability levels of the same mean considerably better for calibrated data: calibration is the only way to bring the probabilities to a sufficiently high level, which is essential for classification purposes.

The result is in agreement with that in [20], where a clear difference was obtained between the sand and grass samples. Other recent studies also point out that the vegetation can be separated from, e.g., built environment using ALS intensity [11], but it seems that the best results will be obtained by combining the intensity with other data (such as range or auxiliary data such as imaging), As to using ALS intensity data alone, at least a better dynamic range would be needed to bring out differences at finer scale, such as those between different stages of vegetation growth. Many more studies are needed to successfully classify, e.g., different vegetation classes.

## 4. Conclusions

We have investigated the effect of radiometric calibration on the accuracy of ALS intensity data combined from separate flights and instruments. In general, the main objective of the absolute radiometric calibration is to produce the intensity values that represent the target reflectance properties and are independent on the measurement system. In this way it would be possible to compare results from different ALS instruments and flights. Our aim was to investigate whether this goal can be achieved, especially with a typical ALS dataset not primarily obtained for intensity calibration purposes.

We find that the calibration with external (naturally available) reference targets improves the accuracy and compatibility of separate intensity data sets and enables land cover classification, but a careful selection of reference targets is necessary. This can be carried out by means of a study of target reflectance and ALS uncalibrated intensity, for which a number of reference targets are needed for each flight, to eliminate the effect of random errors and poor stability typical for ALS intensity data. Therefore, using just one or two a priori selected targets may not produce satisfactory results, especially in case of combining data from separate flights and instruments. We also find that only calibrated data produced probability levels of the same mean that were high enough for classification.

This approach has to be further tested with new ALS campaigns. More accurate results would be possible, if another set of ALS intensity data were available for verification. On the other hand, we used all the available points in the study itself (instead of leaving another group for verification) to improve the statistics. It is not uncommon that a limited number of reference data are available, and there is little time for simultaneous field reference measurements. This is also related to the fact that ALS intensity data are always a side product of the topographic measurement. Therefore this study and its results can be seen as one practical solution to a typical calibration problem. A number of studies are still needed to understand better the radiometric calibration process, its possibilities, and its limitations. This study is one step towards that aim.

## Figures and Tables

**Table 1.** The targets and their backscattered reflectance from ALS and FUJI digital camera. The selected ALS calibration targets for each flight are marked with boldface.

	**Calibrated ALS**	**FUJI**

**April 9, 2008**		

Bodom beach sand	0.20	0.22
Espoonlahti N/artificial grass & sand	0.32	0.34
Espoonlahti/artificial grass	0.43	0.13
Espoonlahti/grass field	0.58	0.59
Espoonlahti swimming pool roof	0.09	0.11
Esport tennis/green sand	0.13	0.28
Kalajärvi school/sand field	0.36	0.39
Kivenlahti marina/sand field	0.41	0.30
Kivenlahti marina/dark gravel	0.23	0.11
Kivenlahti marina/pedestrian	0.24	0.29
Kuninkaanranta beach/wet sand	0.21	0.23
Laurinlahti school/sand field		**0.34**
Luukki golf/bunker sand	0.24	0.27
Luukki golf, green	0.62	0.64
Löfkulla golf, green	0.67	0.65
Löfkulla golf, fairway	0.67	0.56
Maininki school/sand field	0.25	0.28
Olarinniitty/sand field	0.23	0.33
Otaniemi beach path/pedestrian	0.27	0.30
Suomenoja marina/dark gravel	0.09	0.14
Taivallahti tennis/crushed brick	0.31	0.49

**May 12, 2008**		

Eläintarha football field/grass	1.04	0.63
Käpylä football field/grass	0.74	0.60
Pukinmäki football field/grass	0.66	0.64
Tapanila football field/grass		**0.61**

**May 13, 2008**		

Hiekkaharju, sand field	0.50	0.37
Hiekkaharju tenns/red sand	0.55	0.35
Hiekkaharju football field/grass	0.79	0.59
Kallahti school/sand field	0.28	0.40
Kallahti summer beach/sand		**0.34**
Laajasalo football field/grass	0.81	0.57
Mellunmäki football field/grass	0.75	0.61
Myllypuro football field/grass	0.64	0.54
Tuusula Krapi/sand road	0.44	0.40
Tuusula sports, sand field	0.20	0.33
Tuusula, old sand field	0.14	0.25
Tuusula football field/grass	0.54	0.63
Tuusula sports, ash field	0.03	0.09
Vuosaari golf, bunker sand	0.19	0.40
Vuosaari school/sand field	0.23	0.26
Vuosaari sports field shot put	0.35	0.25
Vuosaari sports field/grass	0.88	0.55
Vuosaari sports field/red track	0.45	0.37
